# Microbial community drivers of PK/NRP gene diversity in selected global soils

**DOI:** 10.1186/s40168-019-0692-8

**Published:** 2019-05-22

**Authors:** Chiara Borsetto, Gregory C. A. Amos, Ulisses Nunes da Rocha, Alex L. Mitchell, Robert D. Finn, Rabah Forar Laidi, Carlos Vallin, David A. Pearce, Kevin K. Newsham, Elizabeth M. H. Wellington

**Affiliations:** 1 School of Life Sciences, University of Warwick, Coventry, UK; 2 Department of Environmental Microbiology, Helmholtz Centre for Environmental Research—UFZ, Leipzig, Germany; 3 EMBL-EBI European Bioinformatics Institute, Wellcome Trust Genome Campus, Hinxton, Cambridge, UK; 4 Ecole Normale Superieure (ENS), Alger, Algeria; 5 Centre of Pharmaceutical Chemistry, La Havana, Cuba; 6 Applied Sciences, Faculty of Health and Life Sciences, Northumbria University at Newcastle, Ellison Building, Northumberland Road, Newcastle, NE1 8ST UK; 7 Natural Environment Research Council, British Antarctic Survey, Cambridge, UK; 8 Present addresses: G.C.A.A National Institute for Biological Standards and Control (NIBSC), Potters Bar, UK

**Keywords:** 16S rRNA gene, PKS, NRPS, Natural product, BGCs, Soil, Biogeography, Endemicity, Antarctica

## Abstract

**Background:**

The emergence of antibiotic-resistant pathogens has created an urgent need for novel antimicrobial treatments. Advances in next-generation sequencing have opened new frontiers for discovery programmes for natural products allowing the exploitation of a larger fraction of the microbial community. Polyketide (PK) and non-ribosomal pepetide (NRP) natural products have been reported to be related to compounds with antimicrobial and anticancer activities. We report here a new culture-independent approach to explore bacterial biosynthetic diversity and determine bacterial phyla in the microbial community associated with PK and NRP diversity in selected soils.

**Results:**

Through amplicon sequencing, we explored the microbial diversity (16S rRNA gene) of 13 soils from Antarctica, Africa, Europe and a Caribbean island and correlated this with the amplicon diversity of the adenylation (A) and ketosynthase (KS) domains within functional genes coding for non-ribosomal peptide synthetases (NRPSs) and polyketide synthases (PKSs), which are involved in the production of NRP and PK, respectively. Mantel and Procrustes correlation analyses with microbial taxonomic data identified not only the well-studied phyla *Actinobacteria* and *Proteobacteria*, but also, interestingly, the less biotechnologically exploited phyla *Verrucomicrobia* and *Bacteroidetes*, as potential sources harbouring diverse A and KS domains. Some soils, notably that from Antarctica, provided evidence of endemic diversity, whilst others, such as those from Europe, clustered together. In particular, the majority of the domain reads from Antarctica remained unmatched to known sequences suggesting they could encode enzymes for potentially novel PK and NRP.

**Conclusions:**

The approach presented here highlights potential sources of metabolic novelty in the environment which will be a useful precursor to metagenomic biosynthetic gene cluster mining for PKs and NRPs which could provide leads for new antimicrobial metabolites.

**Electronic supplementary material:**

The online version of this article (10.1186/s40168-019-0692-8) contains supplementary material, which is available to authorized users.

## Background

Environmental bacteria are a source of natural product diversity which formed the basis of early drug development work on antibiotics. Over 80% of all antibiotics deployed in the clinic, as well as ~ 47% of anticancer drugs originate from natural products [1, 2]. Decades of antibiotic misuse, both in the clinic and in agriculture, have led to the worldwide antibiotic crisis with multi-drug-resistant pathogens posing a significant threat to human health [3]. The current dearth of antimicrobial compounds with novel modes of action means that we need to expand our exploration of natural products by investigating the biosynthetic potential of the uncultured soil bacteria through genome mining and metagenomic approaches [4, 5]. Natural products from bacteria have been widely used in human and veterinary medicine and prokaryotic genome analysis has demonstrated that the genes responsible are clustered and many contain non-ribosomal peptide synthetase (NRPS) and polyketide synthase (PKS) enzymes [5]. There is clear evidence that in silico/in vitro combined strategy for identifying NRPS and PKS could provide a rich source of new antimicrobial agents [6].

Genome sequencing has revolutionised natural product discovery with the identification of biosynthetic gene clusters (BGCs) encoding for the production of natural products accounting > 10% of some bacterial genomes such as in the case of *Actinomycetes* [5, 7]. It has been observed that not all the identified BGC sequences could be linked to an antimicrobial product under laboratory conditions due to difficulties with expressing BGCs in culture to a level which facilitates natural product detection and elucidation [8]. Indeed, the production of biologically active natural products in natural environments is a tightly regulated process controlled by a wide range of environmentally activated responses such as *gacA* and *gacS* which control phenazine production in *Pseudomonas* [9] and signalling factors such as γ-butyrolactone and furans in the *Actinomycetales* [10]. Little is known about the factors stimulating the production of such signalling molecules and the natural role of antibiotic biosynthesis in soil has long been debated. However, evidence suggests natural products likely have a role in signalling and protection, with recent studies showing antibiotics playing a key role in antagonism within ant communities and as protective agents in wasps’ nests [11]. Recent genomic evidence has also demonstrated that the less well-characterised *Bacteroidetes*, *Verrucomicrobia* and *Planctomycetes* harbour novel BGCs [12–14], though the functions of these are widely unknown.

Exploring the uncultured soil bacteria for natural products still remains challenging [15]. Efforts have been made to recover single cells from the natural environment. Single cell isolation has now become a key route to understanding the metabolism of uncultured cells, using SiC-Seq to recover the genomes [16]. Such methods are difficult to apply to bacteria intimately associated with soil particles, but large-scale screening efforts combined with iChip technology have uncovered novel genera with bioactive properties [17]. In addition to single cell isolation techniques, analysis of microbial community DNA has enabled the exploitation of metabolic diversity using metagenomic libraries combined with expression screening [18, 19]. However, this approach is challenging if large BGCs such as in the case of natural products need to be recovered. Furthermore, little information was provided in relation to the taxonomic drivers of this diversity [20, 21]. The application of co-occurrence statistics has enabled the linkage of structure to function in microbial communities. Thus, within microbiomes, the ability to understand the importance of diversity in relation to metabolic function has been improved [22].

Two known enzymes for the production of natural products in bacteria are NRPS and PKS. These enzymes are coded by core genes within BGCs which contain all of the information for the biosynthesis of a defined bioactive metabolite, including regulatory elements, transporters and resistant genes [23, 24]. These core genes contain domains that are conserved across all BGCs of that type. The nucleotide identities of such domains can be used to determine phylogenetic relationships between related BGCs, in addition to giving an indication of the natural product encoded. This has been validated at a genomic level and underpins widely used bioinformatics tools such as NaPDoS [25] and anti-SMASH [26]. Recently, we have developed a target assay based on the adenylation (A) domain of the NRPS and the ketosynthase (KS) domain of PKS resulting in amplification of non-conserved regions of these genes that can be used for defining novel metabolic capability [27]. Previous studies using similar targets, focused on soil across the USA, provided evidence that NRP and PK diversity varies with soil type and actinomycete richness [4]. A further survey of Australian soils suggested that both pH and latitude drive BGCs diversity [28]. However, on a global scale across multiple habitats, the taxonomic drivers of BGC diversity are still to be determined.

In collaboration with the British Antarctic Survey (BAS), we became interested in microbial communities within permafrost soils and found preliminary evidence for diversity that could facilitate the exploitation of novel metabolism for bioactive natural products. In particular, BGCs with low similarity to already known clusters belonging to *Actinobacteria* and *Proteobacteria* have previously been isolated and identified through functional metagenomic library screening [27, 29].

The aim of the current study is to understand how differences in microbial community composition drives PK and NRP diversity in a range of soils including those from more extreme environments such as an Antarctic fellfield and the Sahara Desert.

Our experimental design focused on the identification of community structure-function correlations to elucidate the microbial groups driving the diversity in the A and KS domains of the NRPS and PKS biosynthetic genes, respectively. Results presented here shed new light on approaches to discover the potential of unique habitats for PK and NRP BGC diversity.

## Materials and methods

### Soil sample collection and processing

Soil samples were collected from 13 sites across different countries (Algerian Sahara Desert, Mars Oasis in Antarctica, Iceland, Sourhope and Warwick in UK, Tuscany and South Tyrol in Italy, Kilkenny in Ireland, Cayo Blanco and Trinidad in Cuba; Additional file 1: Table S1) under DEFRA licence 51993/194938/3 and sampling permission compliant with national biodiversity legislation. Chemical analyses were performed by YARA Analytical Service, LanCrop Laboratories, Grimbsy, UK. Samples were collected with sterile equipment from the top 10 cm of the soil layer, stored at 4 °C for transport and immediately frozen at −30 °C upon arrival. Large soil samples (300–1000 g) were thawed and three individual samples (0.5 g) were taken from each soil for DNA extraction using the FastDNA® SPIN Kit for soil (MP Biomedicals) according to the manufacturer’s instructions. A Nanodrop spectrophotometer (ThermoFisher) was used to quantify DNA and DNA integrity was confirmed with agarose gel electrophoresis.

### Amplicon library preparation

A total of 117 MiSeq paired-end libraries (2 × 300 bp) were prepared using the Illumina® Nextera XT Library preparation kit for the V3–V4 region of the 16S rRNA gene [30], the A domain of the NRPS gene and the KS domain of the PKS gene [27] (Additional file 1: Table S2). Amplification of the 16S rRNA gene target was performed according to the manufacturer’s instructions (Illumina). Reactions were optimized for A and KS amplicons, with 40 cycles of amplification being used to improve the yield of amplicon DNA recovered (Additional file 1: Table S2).

### Sequence analysis

All amplicon reads are available at the European Nucleotide Archive (study PRJEB11689) and were processed using the EBI Metagenomics analysis pipeline v.3 [31] in order to predict protein coding sequences that could be used for taxonomic analysis (project ERP013097). A custom pipeline composed of PANDAseq [32], USEARCH v.8.1.1861 [33], UPARSE [34], QIIME [35] v.1.9.1 and Kaiju [36] was also used for microbial and functional gene diversity analysis to validate results between different pipelines. In this custom pipeline, paired-end reads were assembled using PANDAseq with a minimum overlap of 10 bp. USEARCH algorithms were then used to de-replicate and sort by size the sequences, discarding singletons. Operational taxonomic units (OTUs) were clustered de novo using the UPARSE algorithm and chimeras were removed based on the prediction of UCHIME. A summary of sequence and observed OTU counts per each dataset is reported in Additional file 1: Tables S3 and S4. A and KS amplicons were initially clustered into OTUs using 95% and 97% similarity but results were comparable (Additional file 1: Table S5) and 97% was selected for all further analyses. The community structure (16S rRNA gene amplicons) was also resolved at 97% OTU similarity. The most abundant sequence per each OTU was selected as the representative sequence. Mitochondrial or chloroplasts sequences were also removed from 16S rRNA gene sequences using the RDP gold database as a reference (in QIIME). Rarefaction was performed using 17,000 sequences for 16S rRNA gene and 8500 or 2600 sequences for A and KS datasets, as cut-offs to obtain OTU tables for further comparative analyses.

### Taxonomic assignment of reads

Taxonomy was assigned to 16S rRNA gene OTUs using the RDP classifier and the Greengenes database.

Raw A and KS reads were processed and analysed with the EBI Metagenomics analysis pipeline, which performs protein prediction using FragGeneScan v1.15 [37]. Additional manual taxonomic annotation of the predicted protein coding sequences derived from A and KS reads were performed using the Unipept [38] software with the NCBI taxonomy classification system. Kaiju web software was also used for the taxonomic annotation of A and KS OTUs [36].

### Annotation of metabolites to A and KS OTUs

Selected A and KS OTUs from the network analysis were associated to potential metabolite families. Blastx against the MIBiG database (version 1.2) [39] was performed with a cut-off expectation value (e-value) of 10^−20^ to prevent misclassification.

### Talent ratio calculation using Integrated Microbial Genomes/Atlas of Biosynthetic Gene Clusters (IMG/ABC) database investigation

In order to determine the biosynthetic potential of communities discovered in the various soil samples, an analysis of specific phyla was done in silico to highlight already existing information on these groups. The IMG/ABC [40] database was manually checked for the number of BGCs and genomes available for each of the main phyla used in the correlation analysis presented in this study. Statistics available on the database were used to retrieve the number of genomes and BGCs (inclusive of PKS, NRPS, saccharides and terpenes) from all available sequenced single isolate genomes deposited in the database (data available on 13/04/2018). BGC evidence (experimentally characterised or predicted only) was also noted. The genetic potential for natural products biosynthesis, here called talent ratio (*T*_R_), was calculated for each phylum as the total number of BGC counts divided by the number of genomes for that specific bacterial group. The *T*_R_ calculation did not consider the different genome average size for each phylum. This indicator was created to investigate theoretical genetic potential for BGC at the phylum level only and not at lower taxonomic ranks.

### Statistical analyses

Statistical analyses were performed using QIIME v. 1.9.1 [35] and R studio v 1.1.456 using the packages Vegan and Phyloseq v 1.16.2 [41]. Alpha diversity was investigated with the Simpson inverse index and part of the beta diversity analysis was based on Bray-Curtis dissimilarity matrices. Differences between rarefied and non-rarefied samples were checked using a paired *t* test (*p* < 0.05) on the Simpson inverse index. OTU networks were created through QIIME, visualized and further developed using Cytoscape [42]. Analysis of similarities (ANOSIM) was performed on groups observed in principal coordinate analysis (PCoA) to test statistically significant difference between groups of samples. The correlation between phyla (as 16S rRNA gene) and the diversity of the two functional genes was investigated using a Mantel correlation between the generated Bray-Curtis dissimilarity matrices for each marker gene, and further explored with a Procrustes superimposition of PCoA plots generated from Bray-Curtis dissimilarity matrices. These analyses were performed between each respective functional gene (A and KS domain) and each separate phylum (filtered 16S rRNA gene by phylum). The phyla selected for correlation analysis had > 1% abundance in at least 20% of the samples representing the core community covering on average 96% of the total bacterial community population in each sample. A correlation-like statistic, Procrustes randomization test (PROTEST) using Monte Carlo simulations (999 permutations) tested the significance of the Procrustes superimposition by evaluating the non-randomness between two configurations. The goodness-of-fit (*M*^2^) value from the PROTEST represented the concordance between the ordinations used in the superimpositions based on the residual sum of squares. The lower the *M*^2^ value, which ranges from 0 to 1, the greater the concordance between the data sets [43]. Procrustes plots were generated with QIIME and visualized using Emperor.

## Results

### A and KS domain diversity across soils

The annotation of translated sequences for A and KS genes obtained through InterPro in the EBI pipeline showed primer specificity for the desired targets. On average, 98% of A reads per sample were assigned to either the AMP-dependent synthetase/ligase domain (IPR000873), the phosphopantetheine binding ACP domain (IPR00081) or the AMP-binding enzyme (IPR002510), while 70–80% of KS reads were assigned either to the thiolase-like (IPR016039) or beta-ketoacyl synthase domains (IPR013030 and IPR013031).

The rarefaction curves demonstrated that for the A and KS domains, diversity was covered by the sequencing with rarefaction not affecting the diversity (A, paired *t* test, *t*(33)=3.67, *p* > 0.05; KS, paired *t* test, *t*(33)=0.997, *p* > 0.05) (Additional file 1: Figure S1). A total of 5834 and 9625 OTUs were observed for the A and KS domains, respectively. Alpha diversity was measured using the Simpson inverse index and ranged across sites from 4.4 to 128.5 for the A domain and from 2.8 to 144 for the KS domain (Additional file 1: Figure S2). The Tuscan soil was significantly more diverse than any of the other soils for both A and KS domains (Tukey’s test, *p* < 0.05).

**Fig. 1.**
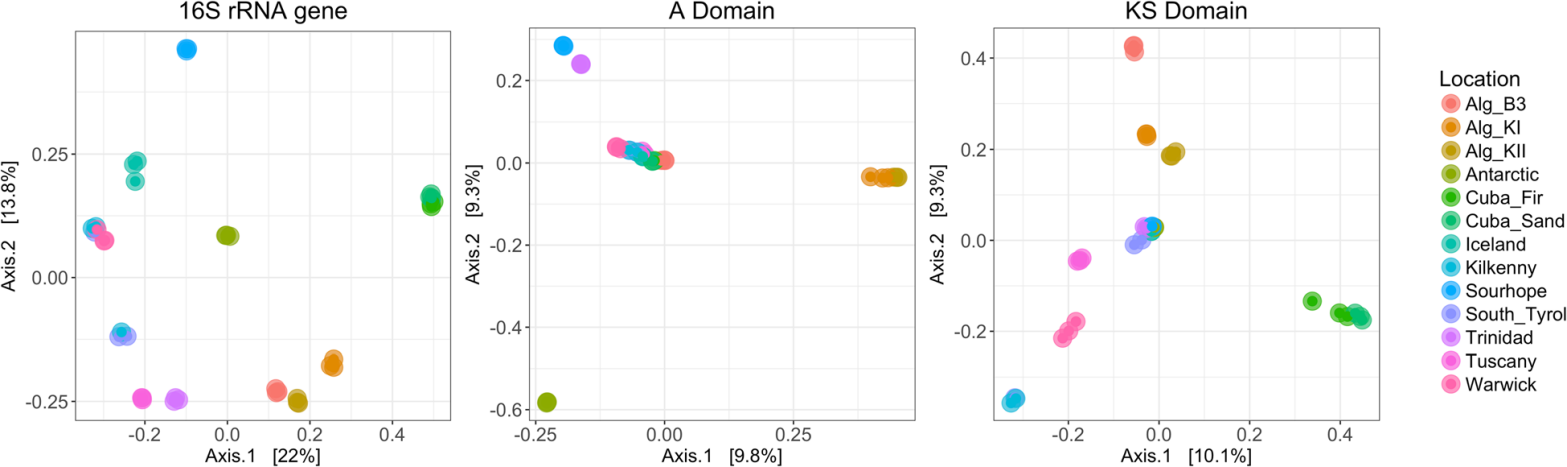
Grouping of soil based on the principal components of diversity in two-dimensions (PCoA). Analysis of A domain, KS domain and 16S rRNA genes based on Bray-Curtis dissimilarity matrices and coloured by location. Alg_KII, Algerian KII; Alg_KI, Algerian KI; Alg_B3, Algerian B3; Cuba-Fir, Cayo Blanco (Fir-Shrub); Cuba-Sand, Cayo Blanco (Shrub)

**Fig. 2.**
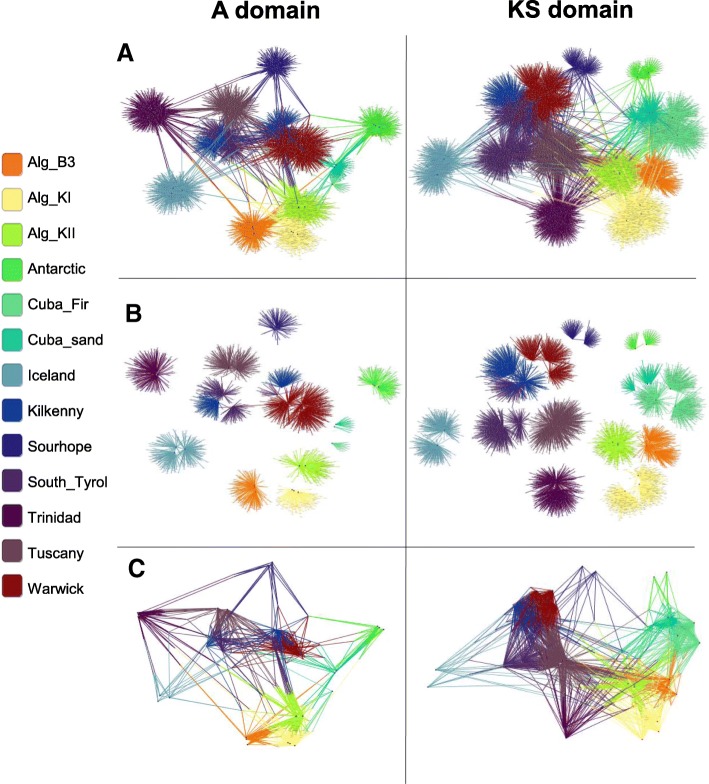
Representation of A and KS domain diversity between samples using OTU networks. Main nodes (black dots) represent soil samples, nodes at the end of edges (grey dots) represent single OTUs. The distance between main nodes is equal to the similarity between samples. Edges connect OTU nodes to sample nodes. A OTU network was constructed for each dataset (A and KS domain separately), and both networks were investigated and represented using a different node degree to represent **a** all OTUs, **b** only location endemic OTUs belonging to each soil sample and **c** only OTUs shared between different locations (not between the same location) (node degree **≥** 4)

The A and KS domain diversity (based on Simpson inverse index) did not show statistically significant Pearson’s correlation to edaphic features such as pH and salinity as electrical conductivity (EC). Linear regression models with one or multiple predictors were also fitted but no statistically significant results were obtained. Beta diversity analysis through PCoA plots of A and KS domains (based on Bray-Curtis dissimilarity matrices) suggested that composition was dependent on geographic location. In particular, for the A domain, European and Cuban samples clustered together, whilst Antarctica, Sourhope, Trinidad and Algeria each formed separate clusters (ANOSIM, *R* = 0.65, *p* = 0.001) (Fig. 1). For the KS domain, Cuban and Algerian soils clustered separately, whilst the European, Antarctic, Icelandic, Trinidad and South Tyrol soils had sequences that showed similarity and grouped together (ANOSIM, *R* = 0.66, *p* = 0.001) (Fig. 1). To visualize shared OTUs between samples and OTUs present only in specific locations, OTU networks of both A and KS OTUs were constructed (Fig. 2). Network separation was based on the number of shared OTU nodes between samples and sites distinctly separated according to geographic location, supporting the results obtained through PCoA plots. A further investigation of the shared and location-specific OTUs across samples allowed the identification of potential areas with endemic A and KS diversity, such as Antarctica and Cuba. These soils harboured lower community diversity than other samples, such as Tuscany, but showed potential endemic A and KS OTUs.

### Microbial community differences across soils

Rarefaction indicated that the bacterial populations (16S rRNA gene) were sampled to sufficient depth (paired *t* test, *t*(38) = 1.128, *p* > 0.05) (Additional file 1: Figure S1).

Alpha diversity with the Simpson inverse index ranged from 22.2 to 404.9, showing significantly lower 16S rRNA gene diversity for the Algerian B3, Cuban and Antarctic samples compared to the other soils (Tukey’s HSD, *p* < 0.05) (Additional file 1: Figure S2). Pearson’s correlation analysis did not show statistically significant correlations between 16S rRNA gene diversity (Simpson inverse index) and the edaphic features pH and salinity (as EC). The analysis of beta diversity through PCoA based on Bray-Curtis dissimilarity matrices identified six distinct groups (ANOSIM, *R* = 0.96, *p* = 0.001): Antarctica, Iceland, Sourhope, Cuba, Algeria and the remaining European soils (Fig. 1). These results suggested differences in the microbial communities according to their geographic locations which were reflected in the microbial community structure at the phylum level (Additional file 1: Figure S3). The mean overall Bray-Curtis similarity value at the genus level between biological replicates was 90.3% suggesting low variability between replicates from the same geographic location.

### Evidence for microbial groups driving A and KS domain diversity

The taxonomic identity of all A and KS OTUs across samples through Kaiju [36] assigned the majority of the OTUs to *Bacteria*. In particular, for the bacterial A and KS domain dataset, the majority of reads were assigned to *Actinobacteria* (average 39% and 26% of the A and KS sequences, respectively) and *Proteobacteria* (average 40% and 24% of the A and KS OTUs) in accordance with the prevalence of these two groups (average 16S rRNA gene relative abundance of 24% and 25%, respectively).

**Fig. 3.**
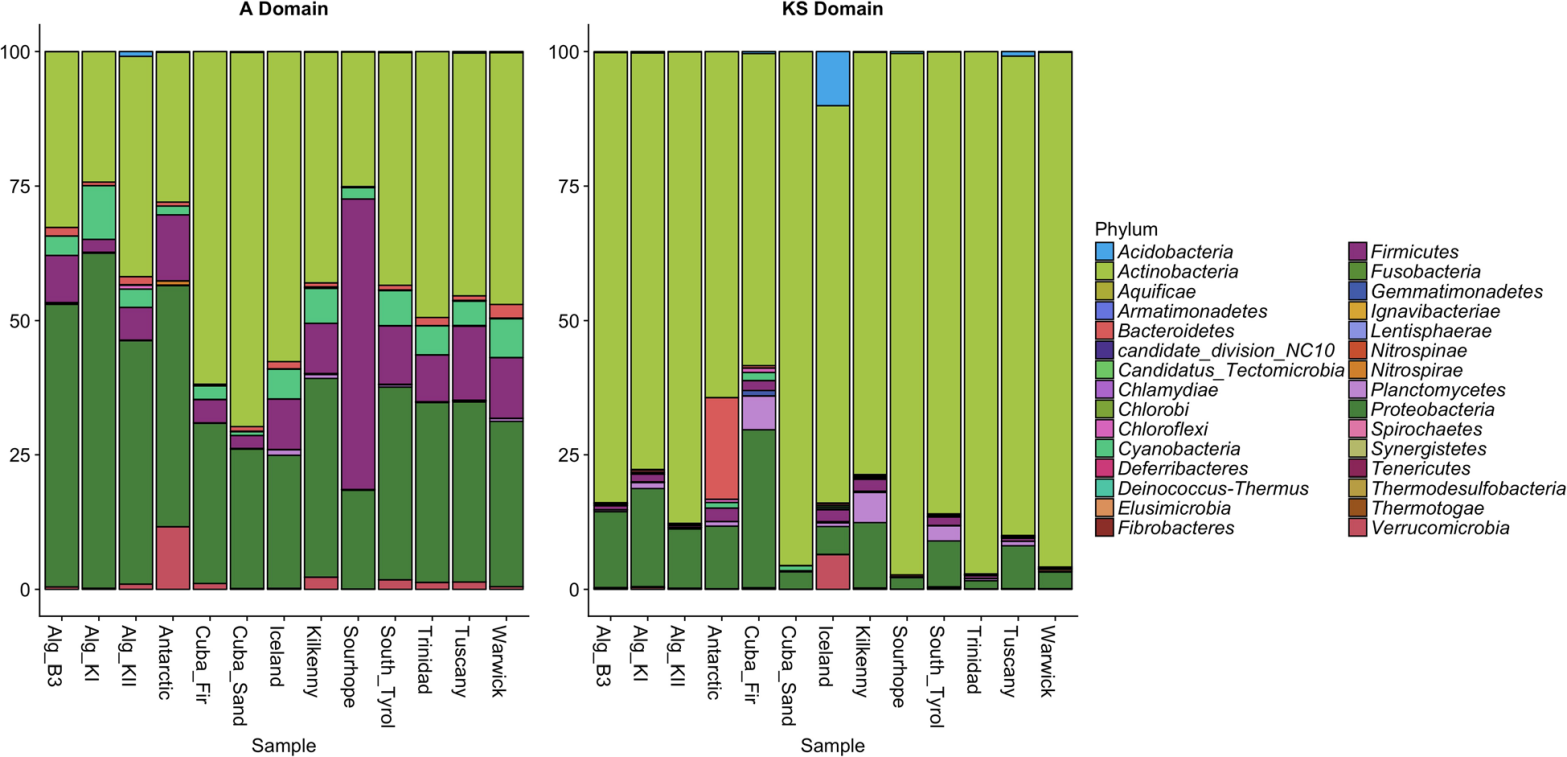
Representation at a phylum level of the taxonomic providence of A and KS domain sequences assigned using the EBI/Unipept pipeline. Phyla with a percentage below 1% in at least 20% of the samples were not individually represented. Bar stats represent mean values of triplicate samples for each site

Identity assignment of A and KS domain amino acid sequences of individual soil samples through EBI/Unipept showed similar results to the Kaiju analysis with an average of 95% and 99% of read of A and KS domain, respectively, assigned to *Bacteria* and the remaining reads were assigned to *Archaea* and *Eukaryota* (Additional file 1: Figure S4)*.* However, this classification highlighted some distinct profiles such as the Antarctic soil which had an average of 10.7% and 16.1% of A and KS reads assigned to *Verrucomicrobia* and *Bacteroidetes* phyla, respectively. Whereas Sourhope showed an average of 53.9% A reads assigned to *Firmicutes* (Fig. 3). The highest abundance of KS reads assigned to the phylum *Acidobacteria* was revealed in the Icelandic soil (average of 6.2%).

According to Mantel correlation analysis, there was a positive correlation between the main phyla of the microbial communities and the biosynthetic diversity (Table 1). The Mantel correlation values for both domains ranged from 0.81 for the well-known producers *Actinobacteria* to the lower score of 0.64 for *Nitrospirae*. In addition, the less well-characterised *Bacteroidetes* phylum had a correlation coefficient of 0.82 and 0.83 for A and KS domains, respectively, whilst the coefficient for the *Verrucomicrobia* were 0.82 for A and 0.79 for KS domain (Table 1).

**Table 1:** Correlation between phyla (16S rRNA gene diversity) and either A or KS domain diversity in all samples

Phylum	R^(***)^	
	A	KS
*Acidobacteria*	0.78534	0.77819
*Actinobacteria*	0.81329	0.80631
*Bacteroidetes*	0.81641	0.82899
*Chloroflexi*	0.78155	0.78091
*Cyanobacteria*	0.75134	0.71288
*Firmicutes*	0.61803	0.6985
*Gemmatimonadetes*	0.81245	0.81914
*Nitrospirae*	0.63576	0.63678
*Planctomycetes*	0.75403	0.74839
*Proteobacteria*	0.77487	0.78704
*Verrucomicrobia*	0.82042	0.79326

The Mantel correlation R values were statistically significant with a *p* value = 0.001 (***)

A number of Procrustes transformation superimpositions were performed on all data and are presented (Additional file 1: Figure S5 and Figure S6) with 16S rRNA gene against A and KS domain data. The correlation with A domains was stronger than for KS, with *M*^2^ of 0.36 (protest, *p* < 0.001) compared to 0.52 (protest, *p* < 0.001). Separate superimposition of each phylum demonstrated the strong relationship between *Actinobacteria* and A domain diversity (*M*^2^ = 0.36, *p* < 0.001). Similarly, for the KS domain, *Bacteroidetes* (*M*^2^ = 0.22, *p* < 0.001) and *Proteobacteria* (*M*^2^ = 0.25, *p* < 0.001) showed a significant goodness of fit with the KS domain superimposition. In particular, this analysis illustrated A and KS domain diversity potentially related to *Actinobacteria*, *Bacteroidetes* and *Verrucomicrobia* in Antarctic soil showing a closer co-location of the datasets on the superimposed PCoA plot.

### Antarctica: a case study for selected functional diversity

As demonstrated by beta diversity and network analysis, the Antarctic soil harboured an endemic community of bacteria and biosynthetic A and KS domains. Taxonomic analysis of shared and location-specific A OTUs recovered from the network analysis (Fig. 2) illustrated that OTUs shared across all sites belonged to *Actinobacteria*, *Proteobacteria* and *Cyanobacteria* species, whilst those endemic to Antarctica also belonged to the *Bacteroidetes* phylum. The same analysis was performed on KS OTUs, whereby only OTUs belonging to the *Actinobacteria* were shared across all sites, whilst many of the endemic OTUs belonged to the PVC (*Planctomycetes*-*Verrucomicrobia*-*Chlamydiae*) group. This suggests that *Actinobacteria*-derived A and KS domains are more widely distributed than those belonging to other phyla.

**Fig. 4.**
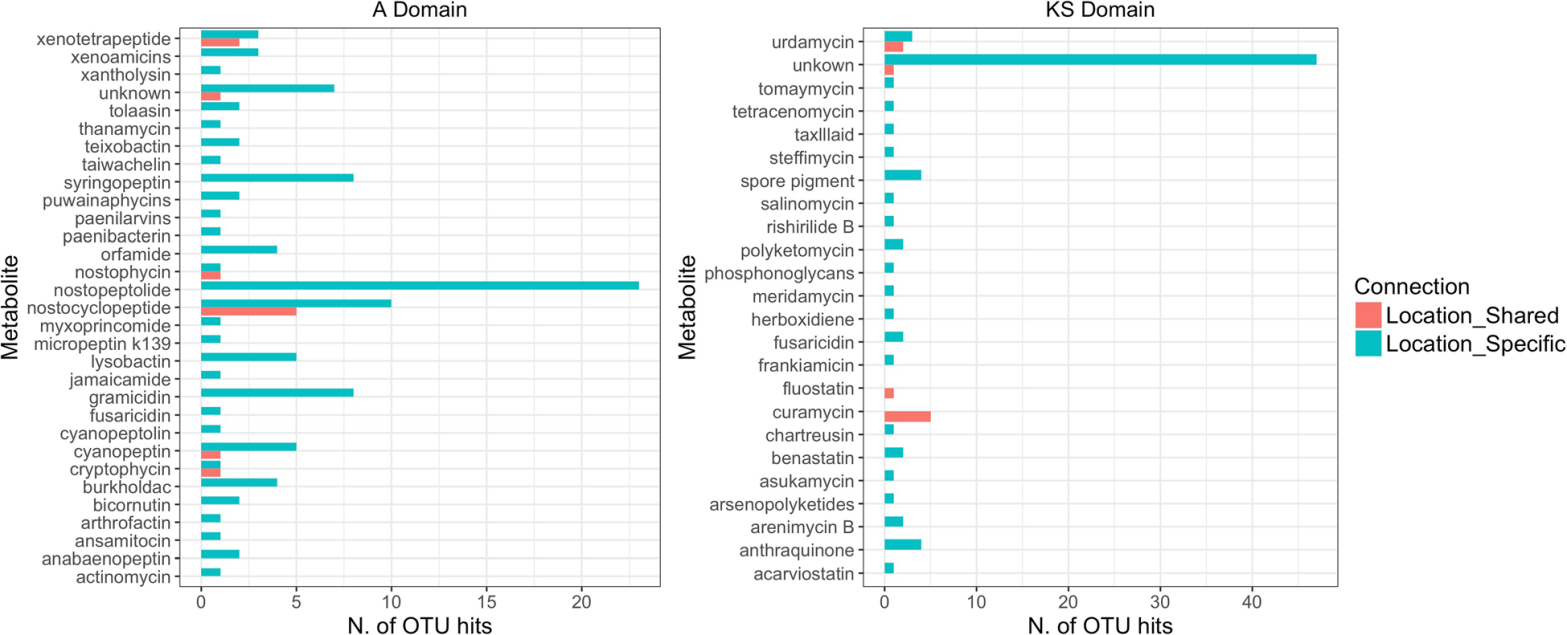
Matched compounds of A and KS location-specific and shared Antarctic OTUs to the MIBiG database. All OTUs from the three Antarctic samples were subject to Blastx analysis for the A and KS domains separately

The assignment of previously characterised compounds (available from the MiBIG database) to Antarctic soil A and KS OTUs indicated that biosynthetic capability for a variety of possible metabolites was present (Fig. 4). Potential metabolites with antimicrobial activity such as teixobactin [17] or antitumor activity as demonstrated for the polyether salinomycin [44] were matched in the database. A large proportion of the total Antarctic endemic OTUs (11% and 59% of A and KS OTUs, respectively) failed to match any of the compounds available in the database suggesting potential for novel endemic metabolites from BGCs.

### Determining the talent ratio for different phyla

The top most abundant phyla across all the soil samples contained some groups poorly represented in genome databases, for example *Verrucomicrobia.* An audit of the IMG/ABC database illustrated the *Actinobacteria* and *Proteobacteria* were by far the most talented bacteria present in the database (talent ratio of 35.83 and 13.02, respectively). A total of 150 genomes were available for *Verrucomicrobia* with 920 predicted BGCs associated with this group. This represents a small fraction in comparison to the most abundant phyla represented in the database such as the *Proteobacteria* with 27,431 genomes available and 357,065 BGCs counted (Table 2). However, the potential for natural product BGCs, expressed as the talent ratio (*T*_R_), showed that *Verrucomicrobia* are comparable to more represented groups such as *Cyanobacteria*, which are better known and characterised for natural product biosynthesis. Similarly, *Bacteroidetes* which only counted 2162 sequenced genomes and 22,885 BGCs have a *T*_R_ of 10.58 which is similar to that of *Firmicutes* (Table 2).

**Table 2:** Counts of biosynthetic gene clusters (BGC) reported on the IMG/ABC database according to phylum and evidence (experimentally proven or predicted only)

Phylum	Genomes available	BGC counts (total)	BGC counts (experimentally proven)	BGC counts (predicted only)	Talent Ratio (*T*_R_)
*Acidobacteria*	100	960	0	960	9.60
*Actinobacteria*	6912	247,650	621	247,029	35.83
*Bacteroidetes*	2163	22,885	2	22,883	10.58
*Chloroflexi*	290	1262	0	1262	4.35
*Cyanobacteria*	1015	8159	60	8099	8.04
*Firmicutes*	15,015	157,654	106	157,548	10.50
*Gemmatimonadetes*	34	69	0	69	2.03
*Nitrospirae*	106	245	0	245	2.31
*Planctomycetes*	193	1343	0	1343	6.96
*Proteobacteria*	27,431	357,065	386	356,878	13.02
*Verrucomicrobia*	150	920	0	920	6.13

Data available on 13/04/2018

Talent ratio (*T*_R_) = BGC counts (total)/Genomes available

## Discussion

The current study provides an insight into the main phyla involved in driving the A and KS domain diversity in a range of soils, focusing on the inter-relationship between structure and function of the microbial community. The geographic segregation of different microbial communities revealed in this study highlights the potential for novel PK and NRP discovery in soils exposed to extreme conditions, such as those of Antarctica, the Algerian Saharan Desert or the pristine Cuban Cayo-Blanco regions. The Antarctic has been the subject of intense study, providing evidence of endemicity amongst the terrestrial metazoans, with endemics also having been identified in cultured and uncultured *Cyanobacteria* and green algae [45]. We posit that the global ubiquity hypothesis [46] is challenged by our observation that metabolic endemism occur in Antarctica but also in Cuba and Algeria. A recent study on an extreme oligotrophic oasis provided strong evidence of high diversity and endemicity for culturable *Actinobacteria* [47]. The phylogenetic uniqueness of *Streptomyces* species recovered from these desert soils provided additional support for migration limitation emphasising the Baas Becking hypothesis of environmental selection*.* We believe that the Antarctic site at Mars Oasis provides similar endemism for PK and NRP metabolite, further implying dispersal limitation.

Correlation analysis between the main phyla and the A and KS domain diversity showed an association between NRPS or PKS genes and less characterised phyla such as *Bacteroidetes* and *Verrucomicrobia*, especially in the Antarctic soils. These two phyla are additional microbial players in metabolite diversity together with the well-known producers *Actinobacteria* [48], *Proteobacteria* [49], *Firmicutes* [50] and *Cyanobacteria* [51]. BGCs from human-associated *Bacteroidetes* were recently identified [52] and a limited number of *Verrucomicrobia* genomes were analysed, identifying possible novel NRPS and PKS genes [12]. The majority of the BGCs reported for both groups have only been predicted and not yet experimentally characterised, but genome mining approaches as well as the results from our study suggest that these groups represent potential reservoirs of novel BGCs. The current study emphasises the potential for less well-characterised phyla being involved in NRP and PK diversity of soil, even though only a small fraction of OTUs were taxonomically matched to these groups. The low number of (annotated) sequences for these phyla in databases may have resulted in fewer matches. Continued sequence analysis combined with experimental characterisation of the predicted clusters will provide an understanding of the biosynthetic potential of these untapped bacterial phyla and the roles in soil ecology of the metabolites that they synthesize.

Antarctic soils harboured A and KS domain diversity unique to this habitat in addition to other reads showed similarities to derivatives of potentially useful antibacterial and anticancer drugs such as teixobactin [17], gramicidin [53], salinomycin [44] and actinomycin [54]. The application of this structure-function relationship analysis has demonstrated the importance of continuing the long-term study of this unique Antarctic habitat.

The identification of variables that affect the microbial community is challenging due to the intrinsic heterogeneity of environmental samples, such as the soil matrix, where different abiotic and biotic variables interact at the micro-scale [55]. However, it has been shown for both microorganisms and macroorganisms that environmental variables and geographic location affect biogeographic patterns of diversity [56–60]. The presence of potentially novel chemical structures in natural products might represent an advantage to microorganisms selecting taxa with different functional potentials to adapt to diverse conditions. Network analysis of A and KS OTUs showed segregation of metabolites forming diversity hotspots, sharing a limited number of OTUs with the other environments. Biogeographical studies have shown that limitation to dispersal, such as physical barriers, do not affect microorganisms but that environmental variables act selectively on the metabolic traits present in the microbial community [61].

## Conclusions

The current study identified unexploited and unexplored taxa, such as *Verrucomicrobia* and *Bacteroidetes*, as potential sources of novel NRP and PK in soils with the implication that geographic isolation was one of the main drivers for microbial community functional diversity. The application of the approach presented in this study will assist in the identification of environments and relevant bacterial groups rich in potentially novel BGCs allowing a more focused targeted approach for drug discovery programmes. The experimental validation of the BGC potential of these less characterised phyla will also promote a better understanding of the role of natural products in the environment.

## Additional files

Additional file 1: **Table S1.** Characteristics and GPS coordinates of soils used in this study. **Table S2.** Primers and conditions used to prepare the amplicon libraries. **Table S3.** Summary of sequence counts per samples and total observed OTU processed with the custom pipeline. **Table S4.** Sequence counts for each sample for all targeted amplicon processed with the custom pipeline. **Table S5.** Correlation between phyla (16S rRNA gene diversity) and either A or KS domain diversity in all samples. **Figure S1.** Rarefaction curves for 16S rRNA gene, A and KS domains diversity. **Figure S2.** Alpha diversity indices for 16S rRNA gene and A and KS domains for each soil sample. **Table S6.** Correlation between Bray Curtis dissimilarity matrixes of each taxonomic level and the original OTU table. **Figure S3.** Community composition at phylum level of each soil sample. **Figure S4.** Representation at the Superkingdom level of the taxonomic providence of A and KS domain sequences. **Figure S5.** Procrustes transformation superimposition of 16S rRNA gene (all phyla or separate) against A domain diversity. **Figure S6.** Procrustes transformation superimposition of 16S rRNA gene (all phyla or separate) against KS domain diversity. (PDF 3191 kb)
