# Supplementary material for: Microbial community drivers of PK/NRP gene diversity in selected global soils
Source: Microbiome. 2019 May 22;7:78. doi: 10.1186/s40168-019-0692-8 (PMC6532259; doi:10.1186/s40168-019-0692-8)
Supplement: Supplementary file 1 — Table S1. Characteristics and GPS coordinates of soils used in this study. Table S2. Primers and conditions used to prepare the amplicon libraries. Table S3. Summary of sequence counts per samples and total observed OTU processed with the custom pipeline. Table S4. Sequence counts for each sample for all targeted amplicon processed with the custom pipeline. Table S5. Correlation between phyla (16S rRNA gene diversity) and either A or KS domain diversity in all samples. Figure S1. Rarefaction curves for 16S rRNA gene, A and KS domains diversity. Figure S2. Alpha diversity indices for 16S rRNA gene and A and KS domains for each soil sample. Table S6. Correlation between Bray Curtis dissimilarity matrixes of each taxonomic level and the original OTU table. Figure S3. Community composition at phylum level of each soil sample. Figure S4. Representation at the Superkingdom level of the taxonomic providence of A and KS domain sequences. Figure S5. Procrustes transformation superimposition of 16S rRNA gene (all phyla or separate) against A domain diversity. Figure S6. Procrustes transformation superimposition of 16S rRNA gene (all phyla or separate) against KS domain diversity. (PDF 3191 kb) [file 40168_2019_692_MOESM1_ESM.pdf]

Additional file 1: Microbial community drivers of PK/NRP gene diversity in selected global soils

Chiara Borsetto[1+], Gregory C.A. Amos[1+], Ulisses Nunes da Rocha[2], Alex L. Mitchell[3], Robert D. Finn[3], Rabah Forar Laidi[4], Carlos Vallin[5], David A. Pearce[6,7], Kevin K. Newsham[7], Elizabeth M.H. Wellington[1*]

[1] University of Warwick, School of Life Sciences, Coventry, UK

[2] Department of Environmental Microbiology, Helmholtz Centre for Environmental Research - UFZ, Leipzig, Germany

[3] EMBL-EBI European Bioinformatics Institute, Wellcome Trust Genome Campus, Hinxton, Cambridge, UK

[4] Ecole Normale Superieure (ENS), Alger, Algeria

[5] Centre of Pharmaceutical Chemistry, La Havana, Cuba

[6] Applied Sciences, Faculty of Health and Life Sciences, Ellison Building, Northumberland Road, Northumbria University at Newcastle, Newcastle, NE1 8ST, UK

[7]Natural Environment Research Council, British Antarctic Survey, Cambridge, UK

Current addresses: G.C.A.A National Institute for Biological Standards and Control (NIBSC), Potters Bar, UK;

[+] Both authors contributed equally to this work

[*] Corresponding author: E.M.H.Wellington@warwick.ac.uk

Soil samples

Table S1. Characteristics and GPS coordinates of soils used in this study.

Soil sample	TC (%)	TOC (%)	TN (%)	pH	EC (µS/cm)	Sand (%)	Silt (%)	Clay (%)	Soil texture	Latitude	Longitude	Country
Algerian B3	1.6	0.8	0.024	8.1	2050	98.49	1.33	0.18	Sand	34.85	5.733333	Algeria
Algerian KI	1.7	0.9	0.040	8.1	2660	95.26	4.14	0.6	Sand	33.3713397	6.8479682	Algeria
Algerian KII	2.1	0.7	0.021	8.1	2280	98.31	1.68	0.01	Sand	33.3713397	6.8479682	Algeria
Antarctica Mars Oasis	0.6	0.6	0.052	8.1	2210	57.3	27.72	14.98	Sandy Loam	-71.886	-68.260775	Antarctica
Cayo Blanco (Fir-Shrub)	11.1	2.7	0.068	8.2	6360	87.72	11.54	0.74	Sand	23.2033	-81.0396	Cuba
Cayo Blanco (Shrub)	9.9	1.2	0.009	8.6	5170	97.03	2.64	0.33	Sand	23.2047	-81.0398	Cuba
Iceland	1.3	1.3	0.089	6.8	2170	79.27	20.31	0.41	Loamy Sand	64.257271	-21.144291	Iceland
Kilkenny	3.3	3.3	0.347	7.1	2050	70.65	25.96	3.39	Sandy Loam	52.88614	-7.50723	Ireland
Sourhope	8.5	8.3	0.729	4.5	82	68.35	25.15	6.50	Sandy Loam	55.47	-2.2313	UK
South Tyrol	10.3	4.2	0.231	7.5	2060	80.78	17.64	1.58	Loamy Sand	46.4982953	11.3547582	Italy
Trinidad	7.6	5.3	0.385	8.0	2580	48.94	39.54	11.52	Sandy Silt Loam	21.7960343	-79.9808143	Cuba
Tuscany	3.2	3.2	0.275	8.0	2240	54.65	33.92	11.43	Sandy Loam	43.5333333	10.71666667	Italy
Warwick	1.4	1.4	0.146	6.9	250	34.41	48.80	16.79	Sandy Silt Loam	52.37622467	-1.569414139	UK

TC = Total Carbon (organic + inorganic) (%); TOC = Total Organic Carbon (%); TN = Total Nitrogen (%); EC = Electrical conductivity (µS/cm);

Amplicon libraries

Table S2. Primers and conditions used to prepare the amplicon libraries.

Primer	Target	Sequence	Annealing temperature (°C)	Amplicon size (bp)	N. amplification cycles	Reference
**16S_F**	V3_V4 16S rRNA	CTACGGGNGGCWGCAG	60	550	25	[1]
**16S_R**	V3_V4 16S rRNA	ACTACHVGGGTATCTAATCC				
**NRPS_F**	A domain	CGCGCGCATGTACTGGACNGGNGAYYT	63	480	40	[2]
**NRPS_R**	A domain	GGAGTGGCCGCCCARNYBRAARAA				
**PKS_F**	KS domain	GGCAACGCCTACCACATGCANGGNYT	61	350	40	[2]
**PKS_R**	KS domain	GGTCCGCGGGACGTARTCNARRTC				

The primers target the phylogenetic marker 16S rRNA (V3-V4 region) and two domains of the functional genes for NRPS (adenylation domain - A) and PKS (ketosynthase domain - KS).

Table S3. Summary of total sequence counts and total observed OTU processed with the custom pipeline.

Target gene	N. of samples	N. of observation	Total count	Counts/sample summary				
				Min	Max	Median	Mean	Std. dev
16S rRNA	39	12236	3422219	17457	222975	79936	87749	53609
NRPS	38	5834	2227137	22	398724	46399	58609	67984
PKS	39	9625	1694806	16	113981	427228	43457	27791

Table S4. Sequence counts for each sample for all targeted amplicon processed with the custom pipeline.

Location	Counts/sample	16S rRNA gene	NRPS	PKS
**Iceland**	S1	24288	95998	52239
**Iceland**	S2	42937	64878	16
**Iceland**	S3	26298	50549	28327
**Trinidad**	S4	17457	46882	53747
**Trinidad**	S5	25563	45917	54
**Trinidad**	S6	30853	69226	51963
**Sourhope**	S7	32207	50096	53768
**Sourhope**	S8	80215	48652	106472
**Sourhope**	S9	80476	49860	49
**Tuscany**	S10	30206	failed	75988
**Tuscany**	S11	27478	21084	39822
**Tuscany**	S12	19819	22021	41516
**South_Tyrol**	S13	44074	5553	30244
**South_Tyrol**	S14	65959	38259	56486
**South_Tyrol**	S15	65750	31044	60355
**Kilkenny**	S16	65933	27321	104394
**Kilkenny**	S17	61958	40558	83
**Kilkenny**	S18	59279	22	25334
**Alg_KII**	S19	129754	398724	32878
**Alg_KII**	S20	105649	75290	38328
**Alg_KII**	S21	84887	99925	21971
**Alg_B3**	S22	69876	24008	55887
**Alg_B3**	S23	222975	26700	43148
**Alg_B3**	S24	114146	52437	46200
**Alg_KI**	S25	90468	89102	26580
**Alg_KI**	S26	61677	76766	37317
**Alg_KI**	S27	62968	83784	63054
**Cuba_Fir**	S28	79936	458	42728
**Cuba_Fir**	S29	113245	423	38141
**Cuba_Fir**	S30	176756	4274	54707
**Antarctic**	S31	132260	118870	12024
**Antarctic**	S32	148898	122156	8589
**Antarctic**	S33	110854	31134	820
**Cuba_Sand**	S34	120526	2620	37851
**Cuba_Sand**	S35	152972	4749	44096
**Cuba_Sand**	S36	209039	1405	68300
**Warwick**	S37	174621	37062	113981
**Warwick**	S38	160953	153342	53902
**Warwick**	S39	99009	115988	73447

Clustering similarity comparison of A and KS domain OTUs in correlation analysis

A and KS domain OTUs were clustered at different level of similarity (95% and 97%) and Mantel correlation analysis with separate phyla were performed with both data sets to determined potential bias related to the clustering similarity threshold used for A and KS domains (Table S5). Results showed the same trend of correlation with both clustering similarity threshold used but higher correlations were detected using 95% clustering similarity. This suggested 97% clustering of the functional genes was more rigorous for determining groups associations.

Table S5. Correlation between phyla (16S rRNA gene diversity) and either A or KS domain diversity in all samples.

Phylum	R (**)			
	A [97%]	A [95%]	KS [97%]	KS [95%]
Acidobacteria	0.78534	0.79574	0.77819	0.80151
Actinobacteria	0.81329	0.8228	0.80631	0.82874
Bacteroidetes	0.81641	0.82478	0.82899	0.84713
Chloroflexi	0.78155	0.79181	0.78091	0.80069
Cyanobacteria	0.75134	0.75196	0.71288	0.71871
Firmicutes	0.61803	0.62822	0.6985	0.70747
Gemmatimonadetes	0.81245	0.81994	0.81914	0.83699
Nitrospirae	0.63576	0.64438	0.63678	0.66246
Planctomycetes	0.75403	0.76414	0.74839	0.77054
Proteobacteria	0.77487	0.78494	0.78704	0.80973
Verrucomicrobia	0.82042	0.83199	0.79326	0.81316

The Mantel correlation R values were statistically significant with a p-value=0.001 (**). The R values were calculated with A and KS domain OTUs clustering at 95% and 97% similarity.

Rarefaction curves

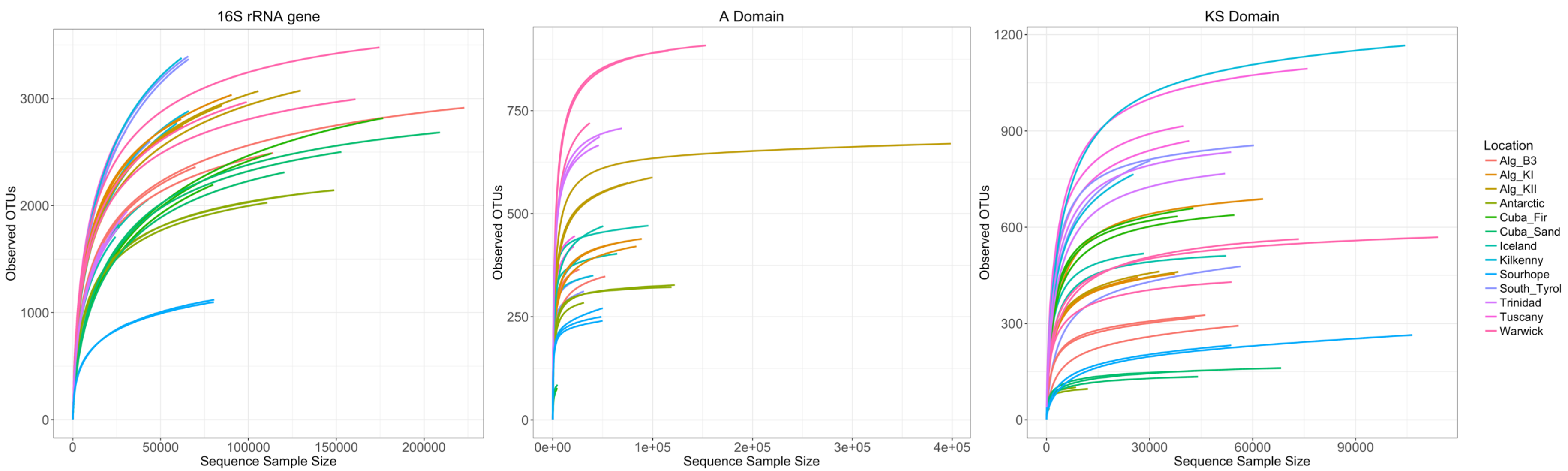

Fig. S1. Rarefaction curves for 16S rRNA gene, A and KS domains diversity. Curves are coloured according to location.

Alpha diversity

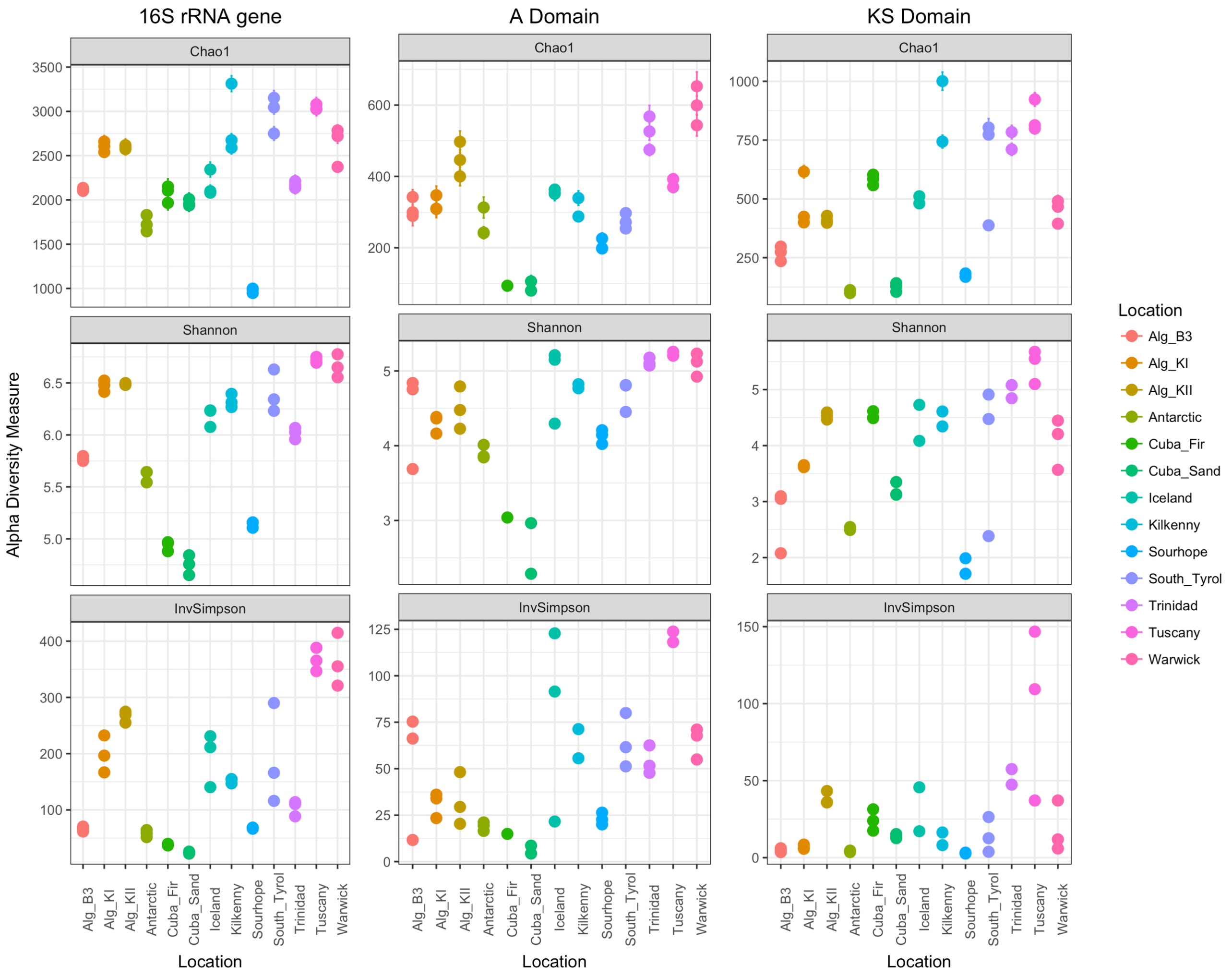

Fig. S2. Alpha diversity indices for 16S rRNA gene and A and KS domains for each soil sample. The Chao1, Shannon and Simpson inverse indices were calculated for each soil sample and reported in different colours according to location.

Community composition analysis

Mantel correlation analysis between each taxonomic level and the original OTU Bray-Curtis dissimilarity matrix was performed to assess the influence of the taxonomic rank to be used for further analysis. Results showed a high correlation at all taxonomic levels suggesting that analysis at all taxonomic levels would be appropriate to achieve comparable results and consequently analogous data interpretation (Table S6).

Table S6. Correlation between Bray Curtis dissimilarity matrixes of each taxonomic level and the original OTU table.

Taxonomic rank	R(**)
Phylum	0.80
Class	0.88
Order	0.90
Family	0.95
Genus	0.96

The Mantel correlation R values were statistically significant with a p-value=0.001 (**).

Community composition

Amplicon sequencing targeting the 16S rRNA gene for all samples (13 sites for a total of 39 samples sequenced), showed differences in the microbial community composition of distinct geographical sites. In general, *Actinobacteria* and *Proteobacteria* were the most abundant phyla (average relative abundance of 24 % and 25 % respectively) (Figure S3). In particular, Antarctic soil had a higher presence of *Cyanobacteria* (average of 28.5 %), *Bacteroidetes* (13 %) and *Verrucomicrobia* (6 %) comparing to the other sites. Cuban soils had a high abundance of *Firmicutes* (39.5 %) and *Bacteroidetes* (6 %) and the lowest representation of *Acidobacteria* (1 %) amongst sites.

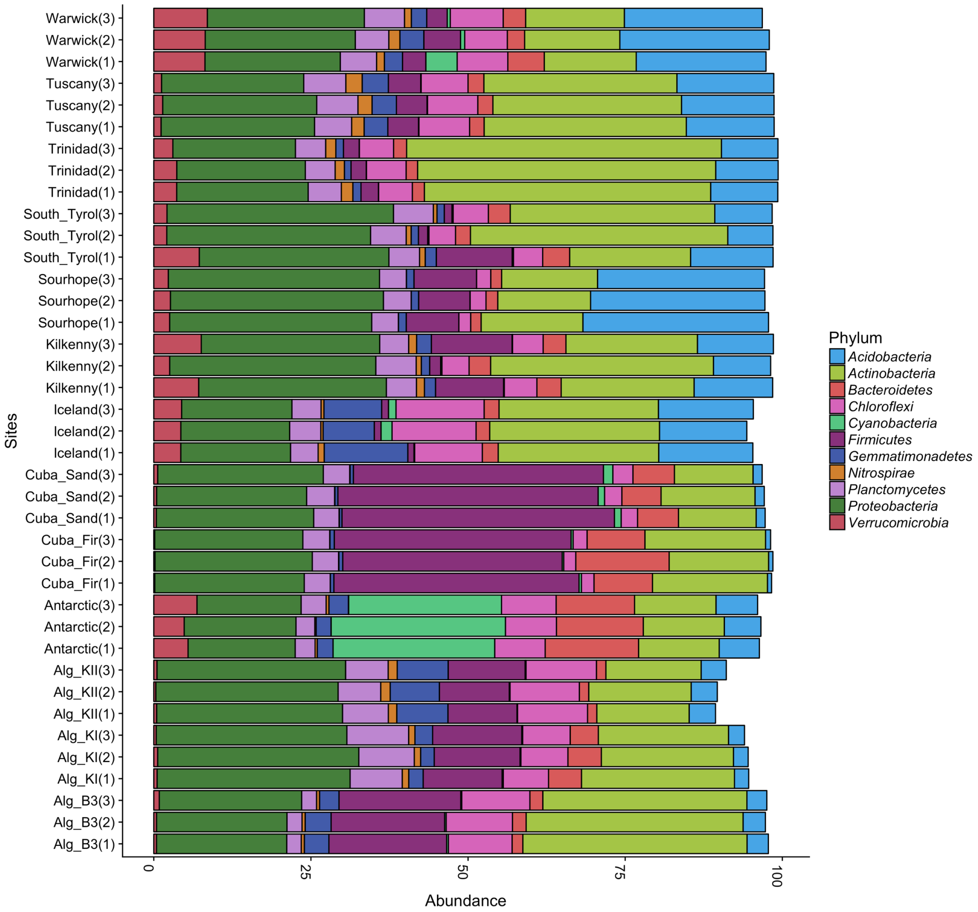

Fig. S3. Community composition at phylum level of each soil sample. Only the phyla which were present at 1% in at least 20% of the samples were reported

Taxonomy assignment of A and K domain OTUs

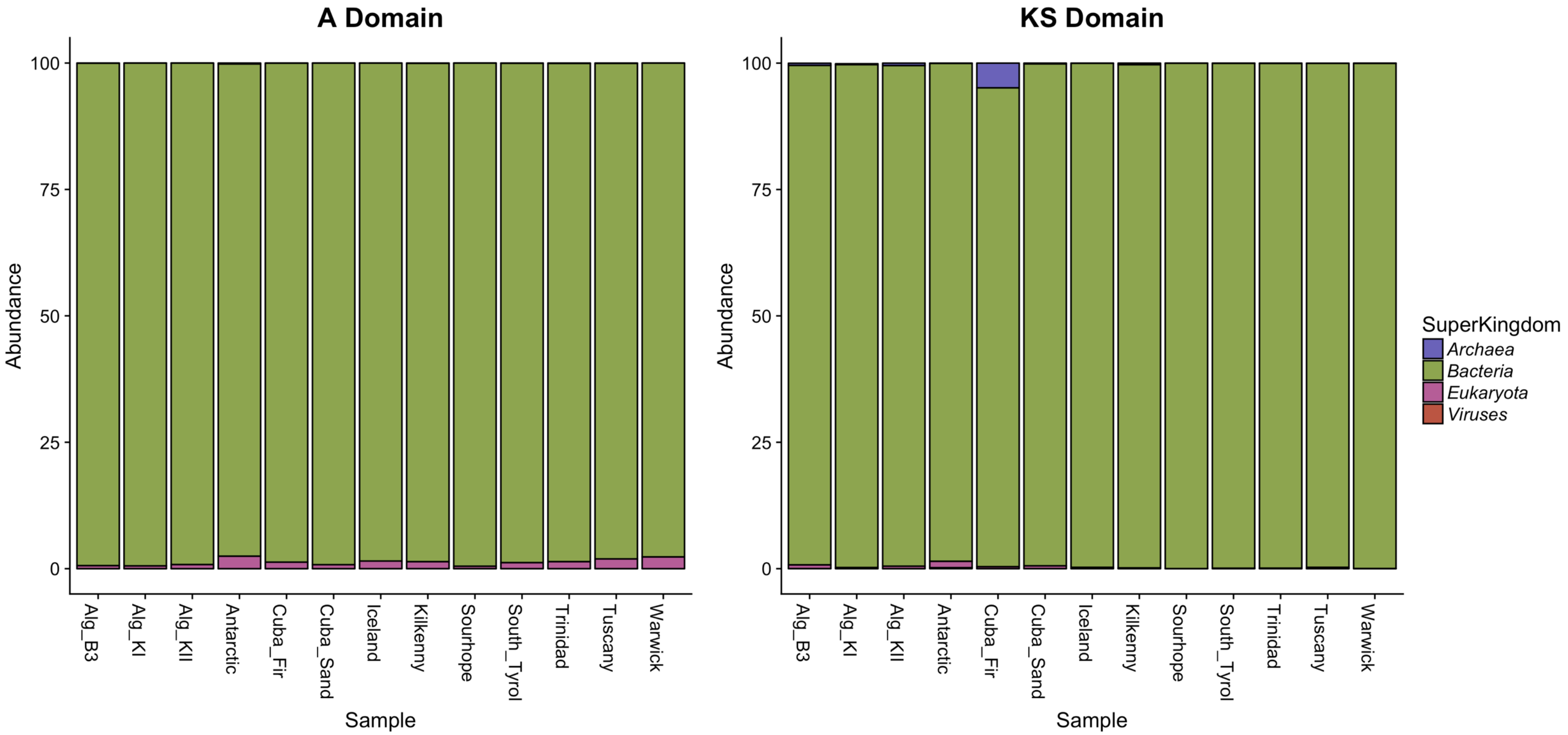

Fig. S4. Representation at the Superkingdom level of the taxonomic providence of A and KS domain sequences. Taxonomic information was assigned to A and KS domain reads using the EBI/Unipept pipeline. Bar stats represent mean values of triplicate samples for each site.

Procrustes plots

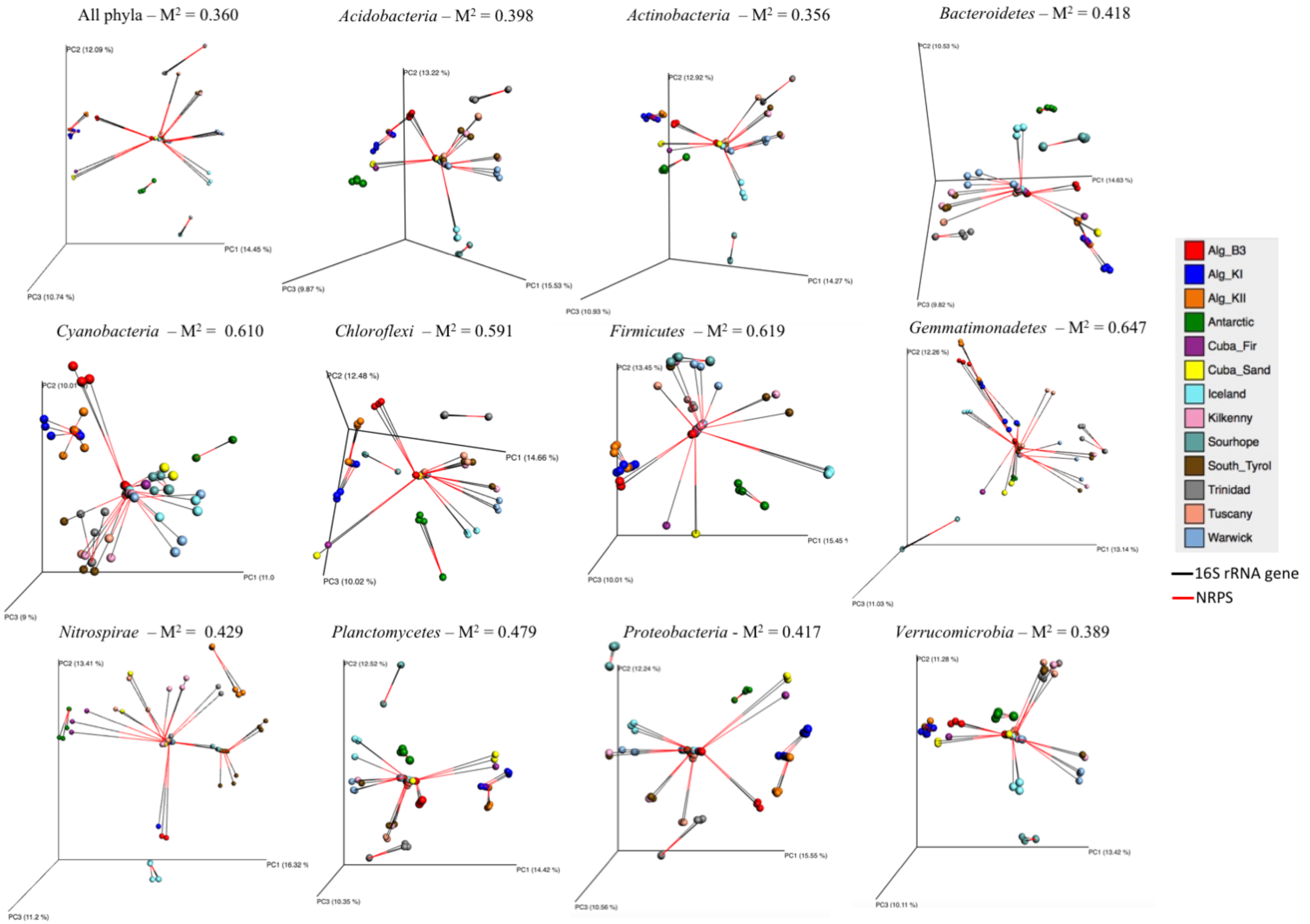

Fig. S5. Procrustes transformation superimposition of 16S rRNA gene (all phyla or separate) against A domain diversity. Locations (nodes) are represented in different colours and edges represent the correlation between the two genes diversity. The M^2 values reported were all significant with a p-value<0.001(**) over 999 permutations.

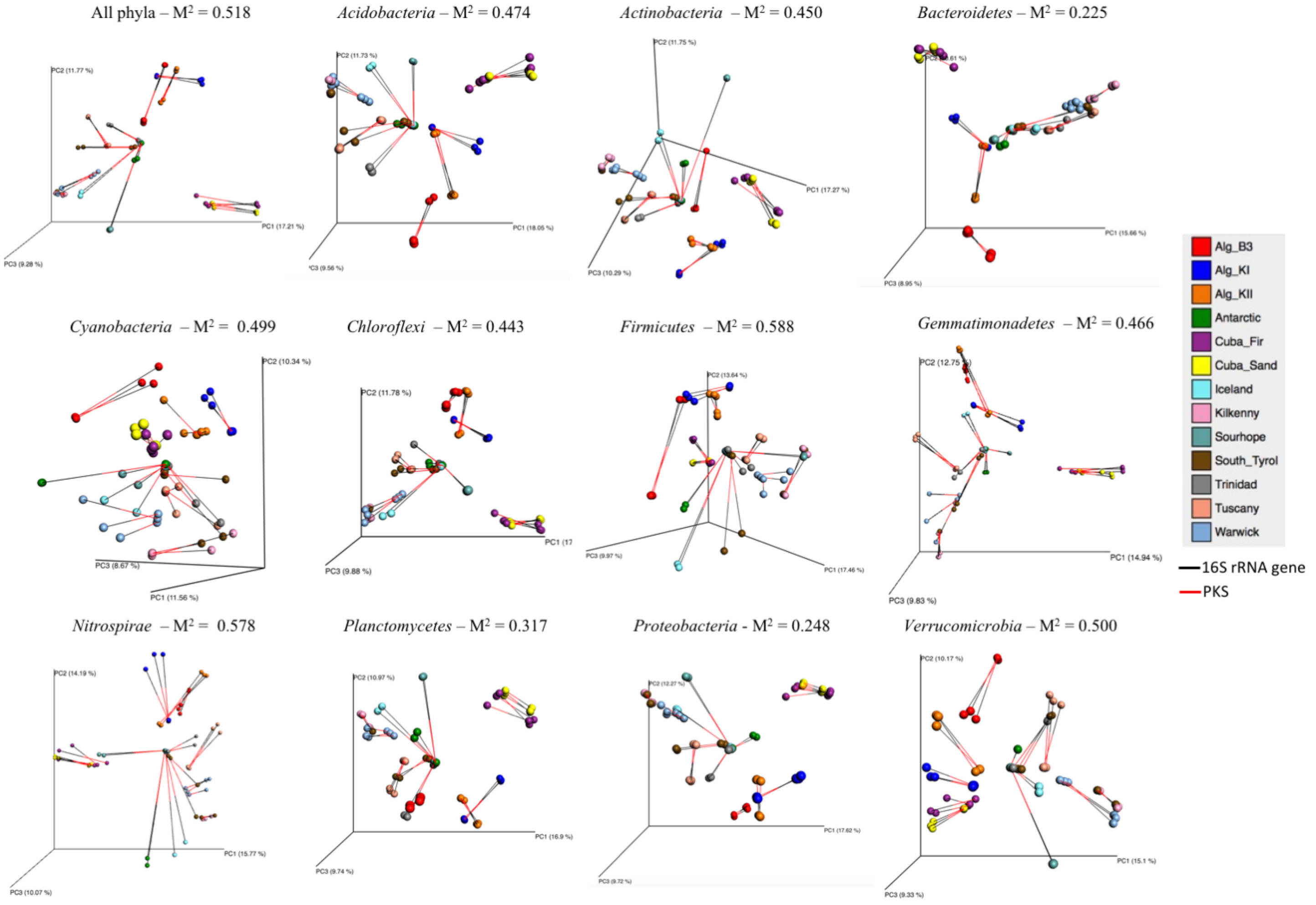

Fig. S6. Procrustes transformation superimposition of 16S rRNA gene (all phyla or separate) against KS domain diversity. Locations (nodes) are represented in different colours and edges represent the correlation between the two genes diversity. The M^2 values reported were all significant with a p-value<0.001(**) over 999 permutations.

References

1. Herlemann DP, Labrenz M, Jurgens K, Bertilsson S, Waniek JJ, Andersson AF: **Transitions in bacterial communities along the 2000 km salinity gradient of the Baltic Sea**. *ISME J* 2011, **5**(10):1571-1579.
2. Amos GCA, Borsetto C, Laskaris P, Krsek M, Berry AE, Newsham KK, Calvo-Bado L, Pearce DA, Vallin C, Wellington EMH: **Designing and implementing an assay for the detection of rare and divergent NRPS and PKS clones in European, Antarctic and Cuban Soils**. *PLoS ONE* 2015, **10**(9):e0138327.
